# Gutmann Donor Theory‐Guided Design of Mononuclear Ionic Cluster for Exceptional N‐Type Thermoelectric Ionogel

**DOI:** 10.1002/advs.202520143

**Published:** 2025-11-21

**Authors:** Bin Chen, Mingxuan Tian, Hongji Wang, Yun Jin, Heng Tang, Xingrui Chen, Shengxue Yu, Changyuan Yan, Wenwei Lei, Taihong Wang

**Affiliations:** ^1^ Hebei Key Laboratory of Applied Chemistry and Hebei Key Laboratory of Heavy Metal Deep‐Remediation in Water and Resource Reuse, School of Environmental and Chemical Engineering Yanshan University Qinhuangdao 066004 China; ^2^ Department of Electrical and Electronic Engineering Southern University of Science and Technology Shenzhen 518055 China

**Keywords:** anion/cation cluster, gutmann donor theory, ion doping, polyacrylate elastomer, ternary thermoelectric ionogel

## Abstract

Quasi‐solid‐state, ionic liquid‐based thermogalvanic gels offer a significant solution for designing ultrasensitive ionic thermopiles due to their exceptional thermopower, superior thermal stability, and high flexibility. Constructing ternary ionogels through ionic doping‐induced ionic aggregates and modulation of ionic transport heat have been proved to be an effective strategy for achieving further enhancement of ionic thermopower. However, the theoretical basis and systematic optimization of ion‐selective doping have not been effectively verified. This study, grounded in the Gutmann donor theory for anion doping, demonstrates four universal configurations of structural reconfiguration in anion/cation clusters within ternary ionogels to progressively enhance anionic transport heat. At 80% relative humidity, this approach achieves a remarkably high negative thermopower of −25.85 mV K^−1^ and a high ionic conductivity of 3.21 mS cm^−1^. Furthermore, this work synergistically complements the Gutmann donor anion‐doping strategy by tailoring the structure of the polyacrylate elastomer matrix and the type of cationic dopant. Ultimately, a wearable device prototype integrating 10 n‐type ion thermoelectric capacitors is demonastrated. This device yields a thermally responsive voltage of 0.774 V (ΔT = 3K), demonstrating promise for designing high‐thermopower ionic thermopiles and harvesting low‐grade thermal energy.

## Graphical abstract

Super‐stretchable and self‐healing polyacrylate ternary ionogel elastomers are used for flexible i‐TE devices. Based on Gutmann donor theory of doped anion, the work proposes a three‐parameter tuning strategy of flexible polymer‐doped anion‐synergistic cation to achieve the systematic design of ionic thermoelectric materials.

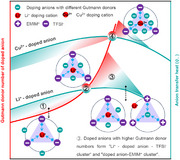

## 1 Introduction

Quasi‐solid, giant thermopower and highly flexible polymer‐based ionic thermoelectric (i‐TE) composites have emerged as attractive candidates for thermoelectric energy storage and temperature difference sensing.^[^ ^1^, ^2^, ^3^, ^4^ ^]^ The core mechanism of i‐TE devices based on the Soret effect can be described as the regular and differential thermal diffusivity of anions and cations in the temperature field, which leads to the accumulation of thermoresponsive potentials and the realization of voltage output.^[^ ^5^, ^6^ ^]^ Theoretically, the thermal response voltage (thermopower, *S_i_*) of i‐TE devices is usually directly related to the difference between the transport heat of cations and anions. And the ion transport heat (*Q_i_*) is mainly regulated by the strong interactions between ion‐ion, ion‐surrounding polymer environment and ion‐electrode. Specifically, using polymers rich in polar functional groups as the backbone, the selection of poly(ethylene oxide) (PEO),^[^ ^7^, ^8^ ^]^ poly(vinylidene fluoride)‐hexafluoropropylene (PVDF‐HFP),^[^ ^9^, ^10^, ^11^, ^12^ ^]^ poly(acrylates) ^[^ ^13^ ^]^ and poly(vinyl alcohol) (PVA) ^[^ ^14^, ^15^ ^]^ to establish strong dipole interactions with the ions has been widely used in the design of i‐TE materials. Similarly, polymers such as cellulose,^[^ ^16^, ^17^, ^18^, ^19^ ^]^ gelatin,^[^ ^20^, ^21^ ^]^ sodium alginate,^[^ ^22^ ^]^ agarose^[^ ^23^ ^]^ and poly(3,4‐ethylenedioxythiophene):poly(styrenesulfonate) (PEDOT: PSS)^[^ ^24^, ^25^ ^]^ have been used to enhance the thermopower of i‐TE materials due to the large number of ionizable polar functional groups on their side chains. Further, the work‐function physical properties of the metal electrode and the abundant oxygen‐containing functional groups on the surface of the carbon electrode material then favor the selective binding of anions and cations, respectively. Thus, reversible bipolar modulation of p‐type and n‐type i‐TE performance is realized.^[^ ^26^, ^27^ ^]^

On the other hand, a new strategy based on the enhancement of *Q_i_* by strong ion‐ion interactions in ionic liquids (IL) has been recently highlighted.^[^ ^28^, ^29^, ^30^ ^]^ This is mainly achieved by the induction of anion and cation clusters by electrolyte additives [lithium tetrafluoroborat(LiBF_4_) and 1‐ethyl‐3‐methylimidazolium chloride (EMIM:Cl)], which in turn increase the anion transport heat (*Q*_−_) and cation transport heat (*Q*_+_), respectively. It is presumed that for the rare n‐type ionic thermoelectric devices with negative thermopower, the increase of *Q*_−_ is not only dependent on the anion clusters constructed by the small‐radius cations, but also is directly related to the closer coordination of the doped anions and small‐radius cations in the electrolyte additives. However, the theoretical mechanism of regulating and optimizing the giant ionic thermopower through selective doping of electrolyte additives (ions) is not clear at this stage, and the related systematic optimization strategies are not fully verified.^[^ ^31^, ^32^ ^]^

The Gutmann donor theory is a widely recognized theoretical basis for measuring differences in the ability of compounds to act as electron donors.^[^ ^33^, ^34^, ^35^ ^]^ In the field of Li^+^/Na^+^ ion batteries, the Gutmann donor number (DN) can be used as a reference to evaluate the strength of the binding ability between electrolyte cations and doped electrolyte anions. Specifically, the Gutmann donor number serves as a quantitative measure of a solvent molecule or ion's capacity to donate electron pairs to a strong Lewis acid. The molecular structural characteristics of the ion, particularly its size and dipole moment, fundamentally determine the magnitude of its DN value. It has been reported that doped anions with high DN can reduce the coordination number of solvent molecules to cations such as Li^+^/Na^+^, thus showing the potential to promote Li^+^/Na^+^ desolvation.^[^ ^36^, ^37^ ^]^ In other words, the higher the DN of an anion, the stronger its coordination ability, thereby facilitating occupation of the inner solvation shell of Li^+^/Na^+^ and forming stable, large anionic clusters tightly enveloped by anions. On the other hand, we know that the relationship between the ionic thermoelectric coefficient and ion transport heat can be expressed as $\alpha =\frac{Q_{i}}{2FT}$. Here, *F*denotes the Faraday constant, *T*represents absolute temperature, and α signifies the thermoelectric coefficient. Furthermore, the transport heat for ion species *i* is given by $Q_{i}=▵H_{i}^{*}-T▵S_{i}^{*}$, where $▵H_{i}^{*}$ represents the transport enthalpy and $▵S_{i}^{*}$ denotes the transport entropy. A larger ion volume typically leads to an increase in both the transport enthalpy $▵H_{i}^{*}$ and the absolute value of the transport entropy $|▵S_{i}^{*}|$. Moreover, the entropy term ($-T▵S_{i}^{*}$) is typically positive. Consequently, an increase in ion volume inevitably leads to a significant rise in both $|▵S_{i}^{*}|$ and the $-T▵S_{i}^{*}$ term, thereby amplifying *Q_i_*. Among these, the contribution of ionic volume to transport entropy ($▵S_{i}^{*}$) represents the most fundamental and potent mechanism by which ionic volume influences *Q_i_*. Entropy change originates from alterations in disorder within the surrounding environment induced by ion movement. Within liquids or flexible polymers, the migration of a large ion triggers substantial rearrangement and disturbance within its extensive solvation shell or interacting polymer segments. This migration process induces a net increase in entropy within the ion's surroundings (i.e., $▵S_{i}^{*}$ is negative). A larger ion volume corresponds to a larger solvation shell and stronger perturbations to polymer chains, thereby causing a greater increase in entropy ($|▵S_{i}^{*}|$). Therefore, the contribution from the entropy term ($-T▵S_{i}^{*}$) becomes more substantial. Inspired by this, it is likely to be a practical solution to analyze and demonstrate the strong ion‐ion interactions to enhance the ion transport heat and amplify the ionic thermopower with the help of the Gutmann donor theory based on anion clusters.

**Figure 1:**
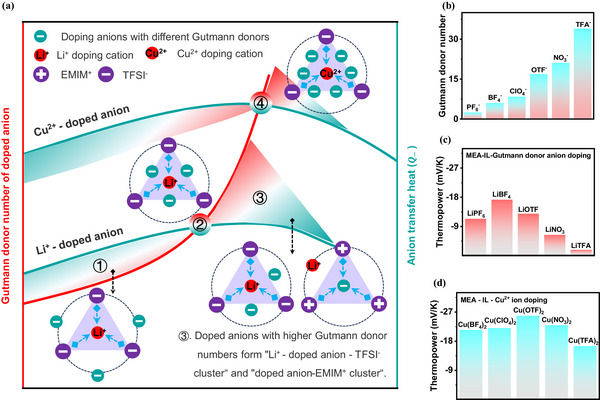
Design principle of n‐type ternary thermoelectric ionogels based on the anion‐doped Gutmann donor theory. a) Anion‐induced conversion of anion/cation cluster structure and the change of anion transport heat in PMEA/Li salt‐EMIM:TFSI and PMEA/Cu salt‐EMIM:TFSI ion gels. b) Gutmann donor number for different doped anions. c) Thermopower of PMEA/Li salt‐EMIM:TFSI ionogels. d) Thermopower of PMEA/Cu salt‐EMIM:TFSI ionogels.

In this study, we introduce a new theory of selective modulation of anion/cation cluster structures based on anion‐doped Gutmann donor theory in ionic thermoelectric materials, leading to simultaneous enhancement of anion transport heat and negative ionic thermopower. We take as a starting point binary ionic liquid‐based acrylate polymer ionogels with impressive stretching, adhesion and self‐healing properties. When a series of Li salts capable of forming close coordination interactions with the ionic liquid are added to form a ternary polymer ionogel (Figure 1b), the negative ionic thermopower first increases and then decreases as the number of Gutmann donors doped with anions gradually increases (Figure 1c). This is mainly due to the gradual interconversion of the anion cluster structure in the ternary ionogels to the conformational interconversion of coexisting anion–cation cluster structures and the gradual weakening of the anion transport heat *Q*_−_ (**Figure** **1**a; Figure S1, Supporting Information). Further, we synchronized the Cu^2+^ cation doping strategy with smaller ionic radius and more concentrated charge density to effectively reverse the coexistence of anion–cation cluster structures. The universality of mononuclear Cu^2+^‐high Gutmann donor anion clusters to enhance the giant negative ionic thermopower was successfully realized (Figure 1d). Practically, a ternary polymer ionogel synthesized in situ from polyethylene glycol methyl ether acrylate ester (PMEA), copper salt [Copper trifluoromethanesulfonate, Cu(OTF)_2_], and IL [1‐ethyl‐3‐methylimidazolidinebis(trifluoromethylsulfonyl)imide), EMIM:TFSI] achieves optimal thermoelectric properties of −25.85 mV K^−1^ at room temperature and 80% RH relative humidity. In addition, our demonstrated ternary polymer ionogels exhibit tunable viscoelasticity and self‐healing properties for easy processing and assembly of modular flexible wearable i‐TE devices. Based on the developed n‐type ionogel and flexible polyimide (PI) substrate materials, 10 serially connected i‐TE devices achieved a thermal response voltage of 0.774 V at a temperature difference of 3 K and exhibited a fast and reproducible temperature response. In conclusion, this result provides a promising insight into the theoretical basis for designing i‐TE materials and highlights the importance of strong ionic interactions‐induced conformational changes of ion clusters and systematic optimization of i‐TE materials.

## 2 Results and Discussion

### 2.1 Synergistic Ionic Association Design for the Binary Ionogels

**Figure 2:**
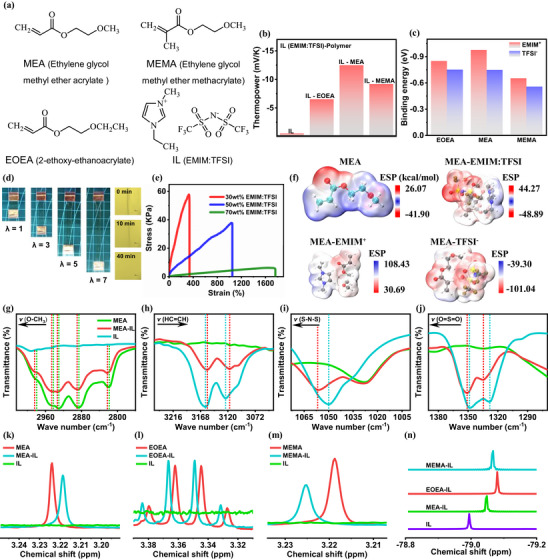
Thermoelectric properties of polyacrylate binary thermoelectric ionogels. a) Chemical structures of MEA, EOEA, MEMA and EMIM:TFSI. b) Thermopower of polyacrylate binary ionogels. c) Binding energies of acrylate monomers with EMIM^+^ cations and TFSI^−^ anions, respectively. d) Optical images of tensile adhesion and self‐healing properties of PMEA/EMIM:TFSI ionogels. e) Tensile stress–strain curves of PMEA/EMIM:TFSI ionogels with different IL contents. f) Electrostatic potential maps. g–j) ATR‐FTIR spectra of ‐OCH_3_ stretching vibrational bands, ‐HC=CH‐ stretching vibrational bands, ‐S‐N‐S‐ antisymmetric bending bands, and ‐O=S=O‐ antisymmetric bending bands in PMEA/EMIM:TFSI ionogels. k–m) H‐NMR spectra corresponding to methoxy in MEA/EMIM:TFSI mixtures, ethoxy in EOEA/EMIM:TFSI mixtures and the terminal methyl group in the MEMA/EMIM:TFSI mixture. n) F‐NMR spectrum attributed to the TFSI^−^ anion in the acrylate monomer and EMIM:TFSI mixture.

The schematic design of the initial binary ionic liquid‐based polymer ionogel is shown in **Figure** **2**a. The ionogel employs the in‐situ copolymer polyacrylate as the solid polymer matrix and EMIM:TFSI as the electrolyte for the ionic liquid (where EMIM:TFSI is loaded at 50 wt%). Polyacrylates amplify the difference of anion/cation transport heat by selectively establishing dipole interactions with anions and cations. For comparison, three structurally similar acrylate monomers were selected, namely, ethylene glycol methyl ether acrylate (MEA), 2‐ethoxy‐ethanoacrylate (EOEA) and ethylene glycol methyl ether methacrylate (MEMA). Pure EMIM:TFSI showed an ionic thermopower of ≈−0.52 mV K^−1^ at 80% RH (Figure 2b; Figure S2, Supporting Information), which is quite consistent with the results reported in the literature.^[^ ^9^ ^]^ The test results showed that compared with the binary ionogels of PEOEA/ EMIM:TFSI (−6.50 mV K^−1^, Figure S3, Supporting Information) and PMEMA/EMIM:TFSI (−9.18 mV K^−1^), the ionic thermopower of PMEA/EMIM:TFSI was the largest at −12.49 mV K^−1^. Apparently, polyacrylates differentially regulated the transport heat of both EMIM^+^ and TFSI^−^ ions. By means of density‐functional theory (DFT), we calculated the interaction forces between the ionic liquids and the three acrylate monomers separately (Figure 2c; Figure S4, Supporting Information). The binding energy between the MEA monomer and the EMIM^+^ cation was calculated to be −0.98 eV, which is much higher than that of the MEA monomer and the TFSI^−^ anion (−0.74 eV). Similarly, EOEA monomer and MEMA monomer were also thermodynamically more favorable for binding EMIM^+^ cations than TFSI^−^ anions. Moreover, the MEA monomer has the maximum value of difference in binding energy with both ions. This may be partly responsible for the fact that the PMEA/EMIM:TFSI binary ionogels exhibited optimal ionic thermopower. As a complementary, the electrostatic potential maps (Figure 2f; Figure S5, Supporting Information) show that the dipole interactions of the MEA monomer with the EMIM^+^ cation (from −41.90 to 44.27 kcal mol^−1^) are stronger than those of the MEA monomer with the TFSI^−^ anion.^[^ ^38^ ^]^ This is consistent with the results of the binding energy tests.

Further, we have also preliminarily evaluated the mechanical properties of PMEA/EMIM:TFSI binary ionic thermoelectric gels. The PMEA/EMIM:TFSI binary ionogel adhering to the ends of the metal electrodes remained firmly adhered to the surface of the metal electrodes even at tensile strains as high as 700% and had a tensile strength of 37.80 KPa (Figure 2d,e). Similarly, the PMEA/EMIM:TFSI binary ionogel also exhibited self‐healing properties within 40 min. We believe that these excellent mechanical properties are beneficial for the application of wearable i‐TE thermoelectric devices and for extending the service life and reducing the maintenance cost.

The Attenuated total reflectance Fourier transform infrared (ATR‐FTIR) spectra further demonstrate the synergistic bonding between the polymer and the ionic liquid (Figure 2g–j; Table S1, Supporting Information). The typical ‐OCH_3_ stretching vibrational bands (2979, 2942, 2930, 2885, and 2818 cm^−1^) of PMEA shifted to the higher wave numbers (2984, 2946, 2933, 2889, and 2822 cm^−1^) upon addition of EMIM:TFSI.^[^ ^13^, ^39^ ^]^ This suggests that the ionic liquid destroys the strong dipole–dipole interactions in the polymer, but enhances the ion‐dipole interactions between the polymer and the ionic liquid. Meanwhile, the ‐HC═CH‐ (3159 cm^−1^) and ‐NC(H)NCH‐ (3123 cm^−1^) telescopic vibrational bands of EMIM^+^ cations are shifted toward short wavelengths. In contrast, the ‐S‐N‐S‐ antisymmetric bending (1050 cm^−1^), ‐O═S═O‐ antisymmetric bending (1348, 1329 cm^−1^) and ‐CF_3_ bending bands (1226, 1167 cm^−1^) attributed to the TFSI^−^ anion are shifted to higher wave numbers. The above results further demonstrate that the strong dipole interaction of EMIM^+^ cations with methoxy in PMEA polymers enhances the n‐type ionic thermopower. In addition, the ATR‐FTIR spectra of PEOEA/EMIM:TFSI and PMEMA/EMIM:TFSI have similar features (Figures S6 and S7 and Table S1, Supporting Information).

In order to further quantify the differences in the thermoelectric properties of the three structurally similar polyacrylates, we further analysed them using the nuclear magnetic resonance (NMR) spectroscopy. The H‐NMR spectra of MEA/EMIM:TFSI, EOEA/EMIM:TFSI, and MEMA/EMIM:TFSI showed that the methoxylation signals of MEA/EMIM:TFSI shifted upward by 0.014 ppm (Figure 2k–m; Figure S8, Supporting Information). In contrast, the ethoxy and other signals of EOEA/EMIM:TFSI and the terminal methyl signals of MEMA/EMIM:TFSI were both shifted downward (Figures S9 and S10, Supporting Information). Theoretically, the signals of protons or functional groups moving toward low field (high ppm) implies strong binding of ions and polymers.^[^ ^30^, ^40^ ^]^ This means that the hydrogen bonding interaction between MEA and TFSI^−^ anion is the weakest. This is the most direct evidence that MEA/EMIM:TFSI exhibits an optimal negative thermopower. Furthermore, the weak interaction between MEA and TFSI^−^ anion was also confirmed using ^19^F‐NMR (Figure 2n). The fluorine signal of MEA/EMIM:TFSI is located at −79.05 ppm, while the fluorine signals of EOEA/ EMIM:TFSI and MEMA/EMIM:TFSI continue to move downward. In fact, replacing the methoxy of MEA with ethoxy (EOEA) from the structure of the polymer would weaken the dipole binding of the ether‐oxygen bond to the EMIM^+^ cation due to the spatial site resistance of ethoxy. In contrast, the introduction of a terminal methyl group (MEMA) into the structure of MEA would increase the hydrogen bond binding to the TFSI^−^ anion. This is the main reason why we initially chose three polyacrylates for comparative analysis. It is clear that the systematic optimization of the polymer structure with due consideration of the synergies between its characteristic functional groups and the ionic liquid anions/cations is central to maximising the negative thermopower.

### 2.2 Regulation of Negative Ionic Thermopower based on Anion‐Doped Gutmann Donor Theory

Theoretically, the thermoelectric properties of i‐TE materials depend on the spontaneous diffusion of ions under thermal gradient (Soret effect). For the equilibrium state of ion thermal diffusion under temperature gradient, the ion thermopower (*S_i_*) can be expressed as^[^ ^24^, ^31^ ^]^:

(1) $$S_{i}=\frac{1}{T}\frac{\sum iq_{i}n_{i}Q_{i}}{\sum iq^{2}n_{i}}$$

where *q_i_*, *n_i_*, and *Q_i_* are the electric charge, concentration, and transport heat of ion species i, respectively. And, *T* is the absolute temperature. Establishing strong ion‐ion interactions and inducing ion aggregation through ion doping, and then regulating the ion transport heat *Q_i_* is an effective method to regulate the ion thermopower. For n‐type ion thermoelectric devices with scarcity and negative thermopower, the anion transport heat *Q_i_* mainly relies on the anion clusters induced by strong coordination interactions between doped cations and ionic liquid anions. On the other hand, the increase of *Q*_−_ is also directly related to the strong interaction between dopant anions and dopant cations. Apparently, the doped anions are also involved in the anion clusters constructed from the dopant cations and ionic liquid anions. And, it is reported in the literature that the tighter the binding of doped anions and doped cations, the more it contributes to the increase of *Q*_−_.^[^ ^29^, ^31^ ^]^ However, will it be possible that the stronger binding energy of the doped anion to the doped cation induces the creation of cationic clusters, which in turn weakens the increased *Q*_−_ of the anionic clusters induced by the doped cation? Based on this speculation, in this section, we construct ternary ionic thermoelectric gels based on PMEA/EMIM:TFSI binary ionogels by doping lithium salts with anions having different Gutmann donors. Among them, the higher the DN of the doped anion, the stronger the coordination ability it possesses, the more likely it is to induce cationic clusters. We believe that evolving a generic configuration for the interconversion of anion/cation cluster structures in the ternary ionogel system based on the theory of Gutmann donors for doped anions is essential for regulating *Q*_−_ and arguing that strong ion‐ion interactions amplify the negative ionic thermopower.

**Figure 3:**
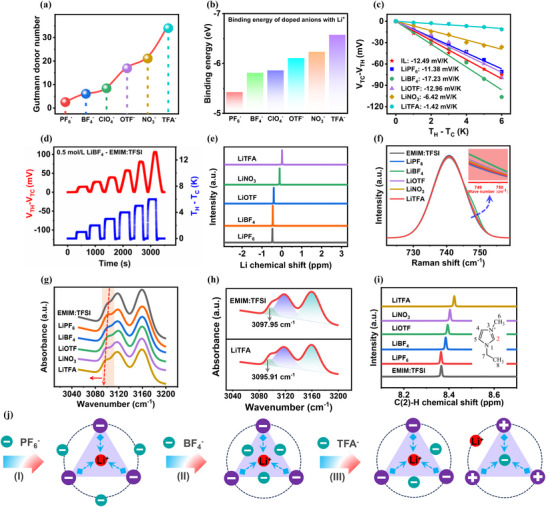
Thermoelectric properties of PMEA/Li salt‐EMIM:TFSI ternary thermoelectric ionogels based on anion‐doped Gutmann donor theory. a) Number of Gutmann donors for different doped anions. b) Binding energies of different doped anions and doped Li^+^ cations. c) Thermopower of PMEA/Li salt‐EMIM:TFSI ionogels. d) Thermal response voltage curve of PMEA/LiBF_4_‐EMIM:TFSI ionogel. e) Li‐NMR spectrum in MEA monomer/Li salt‐EMIM:TFSI mixtures. f) Raman spectra of PMEA/Li salt‐EMIM:TFSI ionogels. g) ATR‐FTIR spectra of PMEA/Li salt‐EMIM:TFSI ionogels. h) ATR‐FTIR spectra of PMEA/EMIM:TFSI and PMEA/LiTFA‐EMIM:TFSI ionogels. i) C(2)‐H‐NMR spectrum in MEA monomer/Li salt‐EMIM:TFSI mixtures. j) Evolution of anion/cation clusters in PMEA/Li salt‐EMIM:TFSI ionogels.

**Figure** **3**a shows the Gutmann donor number (DN) for different doped anions PF_6_^−^ (DN ═ 2.5), BF_4_^−^ (DN ═ 6.03), ClO_4_^−^ (DN ═ 8.4), trifluoromethanesulfonate (OTF^−^, DN ═ 16.9), NO_3_^−^ (DN ═ 21.1) and trifluoroacetate (TFA^−^, DN ═ 34.0).^[^ ^37^, ^41^ ^]^ The binding energy of the doped anions and doped Li^+^ cations was calculated using density functional theory (Figure 3b). As the DN of the doped anion increases, the binding energy of the doped anion and Li^+^ gradually increases. Based on this, ternary ionogels (PMEA/Li salt‐EMIM:TFSI) were prepared by doping lithium salts with anions having different Gutmann donors at 0.5 mol L^−1^ compared to IL (EMIM:TFSI). The thermoelectric properties of the samples were tested, which showed that the ionic thermopower first increased and then decreased with the DN increase of the doped anion (Figure 3c; Figures S11 and S12, Supporting Information). The ternary ionogel of PMEA/LiBF_4_‐EMIM:TFSI obtained a maximum negative ionic thermopower of −17.23 mV K^−1^, and its thermal response voltage had a good linear response at a temperature difference of 6 K (Figure 3d). Apparently, stronger and tighter binding of doped anions and Li^+^ cations does not always contribute to obtaining larger *Q*_−_. This is consistent with our design philosophy. Namely, the binding of doped anions and Li^+^ cations is likely to have an optimal matching value and can ensure the existence of a single anion cluster in the system with Li^+^ cations as the core, thus obtaining the maximum negative ionic thermopower. In other words, if the DN of the doped anion is too large it is likely to produce cation clusters with the doped anion as the ligand core, which leads to an increase in *Q*_+_ and a decrease in the negative ionic thermopower.

To support this argument, we first determined the chemical environment of Li^+^ in Li salt‐doped IL using ^7^Li NMR spectroscopy (Figure 3e). The ^7^Li peak of LiTFA is shifted to the lower field by 0.48 ppm compared to the ^7^Li peak of LiPF_6_^37^. This suggests that the electron density around Li^+^ is reduced in the presence of TFA^−^, implying a stronger binding between Li^+^ and doped anions with high DN numbers. This is in agreement with the results of theoretical calculations. Further, Raman spectroscopy is commonly used to analyze the presence of Li^+^‐anion clusters in ternary ionogels. The bands at 742 and 748 cm^−1^ are attributed to free TFSI^−^ anions and TFSI^−^ anions bound to Li^+^ cations, respectively (Figure 3f; Figure S13, Supporting Information).^[^ ^30^ ^]^ From the figures, we can observe that LiBF_4_ induces more Li^+^‐anion clusters than other anion‐doped Li salts (LiOTF, LiNO_3_, and LiTFA) with stronger Li^+^ binding energy. This indirectly demonstrates that the high DN doped anions are likely to induce the construction of cationic clusters, which in turn compete with the TFSI^−^ anions for the attribution of Li^+^. Echoing this, the TFA^−^ doped PMEA/LiTFA‐EMIM:TFSI ternary ionogel with the largest DN in Figure 3c exhibits an extremely low negative thermopower (−1.42 mV K^−1^) is the most effective experimental proof.

Similarly, we also investigated the evolution of doped anion (high DN)‐cation clusters in ternary ionogels by ATR‐FTIR spectroscopy and ^1^H NMR spectroscopy. For the ATR‐FTIR spectra (3050–3200 cm^−1^), the peak at 3097.95 cm^−1^ was attributed to the C(2)‐H stretching vibration of the imidazole ring of the EMIM^+^ cation (Figure 3g; Figures S14 and S15, Supporting Information).^[^ ^29^ ^]^ The redshift upon addition of LiTFA is a good indication of the formation of hydrogen bonding between the EMIM^+^ cation and the TFA^−^ anion and the appearance of TFA^−^ ‐EMIM^+^/Li^+^ cation clusters (Figure 3h). The formation of cation clusters leads to an increase in *Q*_+_ and promotes thermal diffusion of cations and weakening of the negative thermopower. In addition, the gradual shift of the C(2)‐H hydrogen peak from 8.36 to 8.42 ppm in the ^1^H NMR spectrum (Figure 3i and Figure S16, Supporting Information) is also a good indication of the hydrogen bonding formation between the EMIM^+^ cation and the TFA^−^ anion.

In summary, based on the Gutmann donor theory of anion doped, the conformation of anion/cation cluster structure interconversion in PMEA/Li salt‐EMIM:TFSI ternary ionogels can be divided into three stages (Figure 3j; Figure S1, Supporting Information). Stage I is exemplified by the PMEA/LiPF_6_‐EMIM:TFSI ternary ionogel. PF_6_^−^ has the lowest DN and the weakest binding energy with Li^+^. At this stage, the doped anion of PF_6_^−^ does not participate in the composition of Li^+^‐TFSI^−^ anion clusters, and PF_6_^−^ in the free state does not contribute to *Q*_−_. This is the main reason why the ionic thermopower of the sample does not change much after LiPF_6_ doped. In stage II, the PMEA/LiBF_4_‐EMIM:TFSI ternary ionogel with the increase of doped anion's DN, BF_4_^−^ entered into the Li^+^‐TFSI^−^ anionic cluster structure, and formed the Li^+^‐TFSI^−^/ BF_4_^−^ anionic cluster structure with a larger *Q*_−_. In stage III, the PMEA/LiTFA‐EMIM:TFSI ternary ionogel was taken as an example. The TFA^−^ anion has the largest DN number and form a cationic cluster with partially TFA^−^ as the ligand core. Moreover, part of Li^+^ in the Li^+^‐anion cluster structure tends to participate in the formation of TFA^−^‐Li^+^ cation clusters. At this time, both Li^+^‐TFSI^−^/ TFA^−^ anionic cluster and TFA^−^‐EMIM^+^/Li^+^ cationic cluster structures both exist in the PMEA/LiTFA‐EMIM:TFSI ternary ionogel. This is the main reason for the sharp decrease in the negative thermopower of PMEA/LiTFA‐EMIM:TFSI ternary ionogel.

### 2.3 Cation Doping Pervasively Enhances Negative Ionic Thermopower

**Figure 4:**
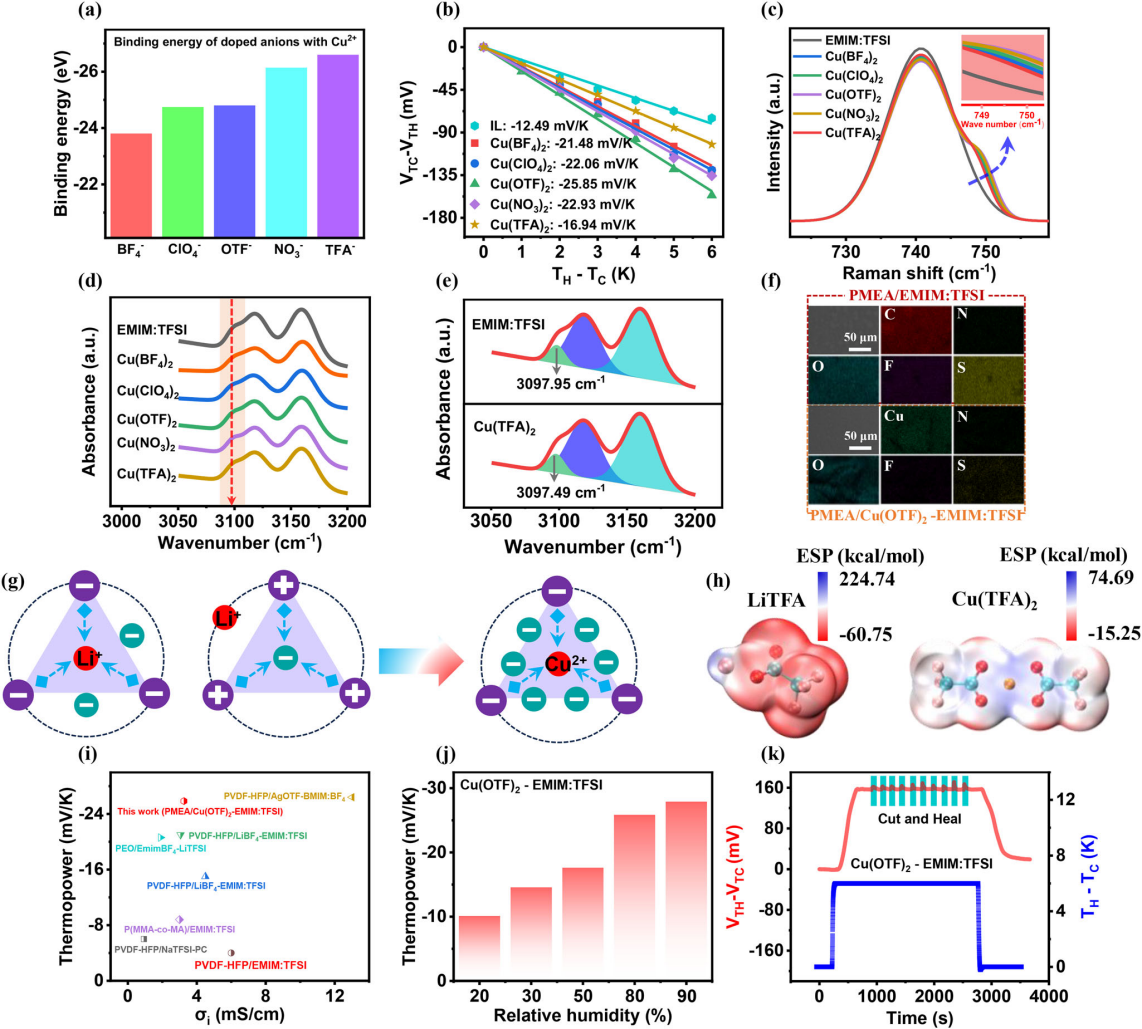
Thermoelectric properties of PMEA/Cu salt‐EMIM:TFSI ternary thermoelectric ionogels based on Cu^2+^ cation doping. a) Binding energies of different doped anions and doped Cu^2+^ cations. b) Thermopower of PMEA/Cu salt‐EMIM:TFSI ionogels. c) Raman spectra of PMEA/Cu salt‐EMIM:TFSI ionogels. d) ATR‐FTIR spectra of PMEA/Cu salt‐EMIM:TFSI ionogels. e) ATR‐FTIR spectra of PMEA/EMIM:TFSI and PMEA/Cu(TFA)_2_‐EMIM:TFSI ionogels. f) Elemental mapping results. scale bars, 50 µm. g) Conformations of ion clusters in PMEA/Cu salt‐EMIM:TFSI ionogels. h) Electrostatic potential diagram. i) Comparison of thermoelectric properties of PMEA/Cu(OTF)_2_‐EMIM:TFSI ionogel in this study with previously reported n‐type ionogels.^[^ ^9^, ^10^, ^13^, ^28^, ^29^, ^30^, ^31^ ^]^ j) Humidity‐modulated thermopower of PMEA/Cu(OTF)_2_‐EMIM:TFSI ionogels. k) Change in thermal response voltage after multiple cut‐heal cycles.

The decrease in thermopower of PMEA/LiTFA‐EMIM:TFSI ternary ionogel can be attributed to the TFA^−^ anion with high Gutmann DN induce TFA^−^ ‐EMIM^+^/Li^+^ cation clusters. In other words, if the doped cation can effectively lock the doped anions with high DN, it can be ensured that only mononuclear anion clusters are present in the ionogels will achieve the maximisation of the negative ionic thermopower. It is well known that copper ions (Cu^2+^) have a higher ionic charge and smaller ionic radius (ionic radius ≈73 pm) compared to the doped Li^+^ cations (ionic radius ≈76 pm). This confers a tighter bound and stronger binding of Cu^2+^ and doped anions of different DN. DFT calculations showed that the binding energy of Cu^2+^ with different doped anions (from −23.8 to −26.6 eV) was much higher than those of Li^+^ ‐doped anions (from −5.4 to −6.6 eV) (**Figure** **4**a). Therefore, we investigated the thermoelectric properties of PMEA/Cu salt‐EMIM:TFSI ternary ionogels and aim to achieve further enhancement of Cu^2+^ cation‐induced mononuclear anion clusters and *Q*_−_.

Specifically, the thermoelectric properties of PMEA/Cu salt‐EMIM:TFSI ternary ionogels were all substantially improved (Figure 4b; Figures S17 and S18, Supporting Information), and the samples' thermopowers were all much larger than those of the PMEA/EMIM:TFSI binary ionogels. Impressively, PMEA/Cu(TFA)_2_ ‐EMIM:TFSI breakthrough achieved a thermopower of −16.94 mV K^−1^, much higher than that of PMEA/LiTFA‐EMIM:TFSI (−1.42 mV K^−1^). Apparently, the doping of Cu^2+^ cations is universal for the thermopower's enhancement of the ternary ionogels. This is in agreement with our experimental expectations. In addition, the ionic thermopower follows the same trend of increasing and then decreasing as the DN of the doped anion increases. And, the maximum negative ionic thermopower of −25.85 mV K^−1^ was obtained for PMEA/Cu(OTF)_2_ ‐EMIM:TFSI. The above results preliminarily indicate that Cu^2+^ cation can effectively achieve locking up the high DN doped anions, but do not completely eliminate the presence of cationic clusters. Our experiments emphasise the importance of the matching of doped cations and anions to achieve the negative ionic thermopower.

Further, we also investigated the evolution of Cu^2+^ cation‐induced anion clusters in PMEA/Cu salt‐EMIM:TFSI ternary ionogels by Raman spectroscopy and ATR‐FTIR spectroscopy. In Raman spectroscopy, we can clearly observe the TFSI^−^ anions closely bound to Cu^2+^ cations at 748 cm^−1^ (Figure 4c; Figure S19, Supporting Information). Moreover, the binding of Cu^2+^ cations to TFSI^−^ anions is stronger than that of Li^+^ cations to TFSI^−^ anions (Figure 2f). On the one hand, it favors the binding of Cu^2+^ cations with more anions (TFSI^−^ and doped anions) to form anion clusters. On the other hand, this also favors the high DN doped anions being firmly locked and reduces the overall percentage of cation clusters in the ionogel. By performing relative integration on Raman spectra, we further estimated the percentage of TFSI‐ anions bound to Cu^2+^ (Figure S20, Supporting Information). Among the samples, the PMEA/Cu(OTF)_2_‐EMIM:TFSI ionogel exhibited the highest content of anionic clusters formed by TFSI^−^ anions and Cu^2+^. These integration results are consistent with the trend observed in the variation of ionic thermopower. In particular, the microregion magnified view of the Raman spectra also shows that somewhat more TFSI^−^ anions are bound to Cu^2+^ cations in PMEA/Cu(OTF)_2_‐EMIM:TFSI compared to PMEA/Cu(TFA)_2_‐EMIM:TFSI. This is most likely due to the fact that the high DN of the doped anion TFA‐ still inevitably induces some of the cation clusters. However, the Cu^2+^‐TFSI^−^/TFA^−^ anion clusters were still the main component in the PMEA/Cu(TFA)_2_‐EMIM:TFSI ternary ionogels anyway. This was also demonstrated by ATR‐FTIR spectroscopy, in which the C(2)‐H stretching vibration of the imidazolium ring of the EMIM^+^ cation underwent only a very slight redshift (Figure 4d,e; Figures S21 and S22, Supporting Information). Meanwhile, to more directly observe the thermal diffusion process of anion clusters under a temperature gradient, we performed in situ Raman spectroscopy on the PMEA/Cu(OTF)_2_‐EMIM:TFSI ternary thermoelectric ionogel. As shown in Figure S23 (Supporting Information), the laser source was focused at fixed positions on the hot and cold ends during measurement. The peak intensities of the free TFSI^−^ anion (742 cm^−1^) and the TFSI^−^ anion bound to Cu^2+^ cations (748 cm^−1^) gradually decreased at the hot end of the ionogel with prolonged heating time, while the peak intensities at the cold end gradually increased. We know that ions with greater mobility tend to accumulate at the cold end. This is the most direct manifestation of the PMEA/Cu(OTF)_2_‐EMIM:TFSI ternary thermoelectric ionogel exhibiting a negative thermopower and n‐type ion‐thermoelectric behavior. In addition, we analysed the elemental distribution in PMEA/Cu(OTF)_2_‐EMIM:TFSI (Figure 4f). The elemental mapping results showed that the C, O, F, N, S, and Cu elements were uniformly distributed in the gel, demonstrating that Cu(OTF)_2_ and EMIM:TFSI were well‐dispersed in the PMEA matrix.

Overall, the modulation of the negative thermopower in ternary ionogels can be attributed to two specific contributions. In the first aspect, doped anions with different Gutmann DN are involved in the structure of the anion clusters. This allows the anion clusters to maximise the regulation of the common *Q*_−_ of the TFSI^−^ and dopant anions. On the other hand, the higher ionic charge and smaller ionic radius of the Cu^2+^ cation constructed extremely strong anion clusters. This allows us to obtain extremely good negative ionic thermopower materials even in the face of higher DN doped anions (Figure 4g). This is also reflected in the electrostatic potential plot, where the Cu(TFA)_2_ model exhibits a smaller overall electrostatic potential, which reflects the great energy of the Cu^2+^ cation for binding the TFA^−^ anion (Figure 4h). In addition, in comparison with the existing relevant literature, our prepared PMEA/Cu(OTF)_2_‐EMIM:TFSI ternary ionogel have negative thermopower comparable to the highest values currently available for n‐type ionogels (Figure 4i; Table. S2, Supporting Information). Then, we also tested the short circuit current‐open circuit voltage and corresponding output power of the i‐TEC constructed from the PMEA/Cu(OTF)_2_‐EMIM:TFSI ternary thermoelectric ionogel (Figure S24, Supporting Information). The test results indicate that as the temperature gradient increases from 2 K to 6 K, the open‐circuit voltage and short‐circuit current gradually increased from 48.12 mV and 3.65 µA to 156.56 mV and 12.07 µA, respectively. And, the maximum output power increased significantly from 44.47 to 234.84 nW, exhibiting a near‐quadratic relationship with the open‐circuit voltage.

Moreover, the humidity also affects the thermoelectric properties of the samples, and the higher the relative humidity, the higher the negative ionic thermopower (Figure 4j). This is primarily due to the methoxy functional groups of the hydrophobic polymer PMEA forming partial intermediate water. Under humid conditions, moisture absorbed by PMEA penetrates into the polymer backbone, improving ion transport pathways by reducing the polymer's crystallinity. This increases ionic conductivity and allows the initial disparity in thermal transport rates between cations and anions to be further amplified, thereby enhancing the ionic thermopower. We note that even under humidity conditions of 50% RH, our thermoelectric device still exhibits a thermopower of −17.63 mV K^−1^ and an ionic conductivity of 2.97 mS cm^−1^. This places it among the most advanced n‐type ionic thermoelectric devices. The PMEA/Cu(OTF)_2_‐EMIM:TFSI ternary ionogel is also self‐healing, and the thermal response voltage is able to revert back to the initial value after several cutting and self‐repairing cycles (Figure 4k). In fact, the Debye forces between the electronegative oxygen atoms on the ester and methoxy groups and the electropositive carbon chains in the PMEA polymer structure drive the self‐healing properties of PMEA‐based ternary ion gels (Figure S25, Supporting Information). Based on molecular dynamics simulations, charge distribution results for MEA monomers clearly indicate that electronegative oxygen atoms carry more concentrated negative formal charges. Although individual Debye forces are typically considered weak (<10 kJ mol^−1^), the synergistic effect of multiple Debye forces can simultaneously provide dynamic yet extremely strong bonding. We additionally tested the recovery rate of the PMEA/Cu(OTF)_2_‐EMIM:TFSI ternary thermoelectric ionogel after 10 self‐healing cycles under 0–500% tensile strain. Test results indicate that the thermopower recovery rates after 500% tensile strain and the 10th self‐healing cycle remain at 98.18% and 96.25% of their original values, respectively. These findings demonstrate that the microscopic ionic channels remain intact even after multiple tensile cycles and self‐healing, thereby preserving the ionic thermoelectric properties. And we have also tested the thermoelectric properties of PMEA/Cu(OTF)_2_‐BMIM:TFSI and PMEA/Cu(OTF)_2_‐EMIM:BF_4_ ternary thermoelectric ionogels by altering the type of IL (Figure S26, Supporting Information). The results indicate that both PMEA‐BMIM:TFSI and PMEA‐EMIM:BF_4_ binary thermoelectric ionogels exhibit limited positive ionic thermopower. In stark contrast, ternary thermoelectric ionogels incorporating Cu(OTF)_2_ not only achieved a reversal of the ionic thermopower but also exhibited substantial negative ionic thermopower (‐23.1 and −12.5 mV K^−1^). Based on these experimental results, we have reason to believe that regulating anion cluster configurations to achieve negative ionic thermopower represents a promising solution.

### 2.4 Ionic Thermoelectric Capacitors (i‐TEC) and Modular i‐TEC Performance

**Figure 5:**
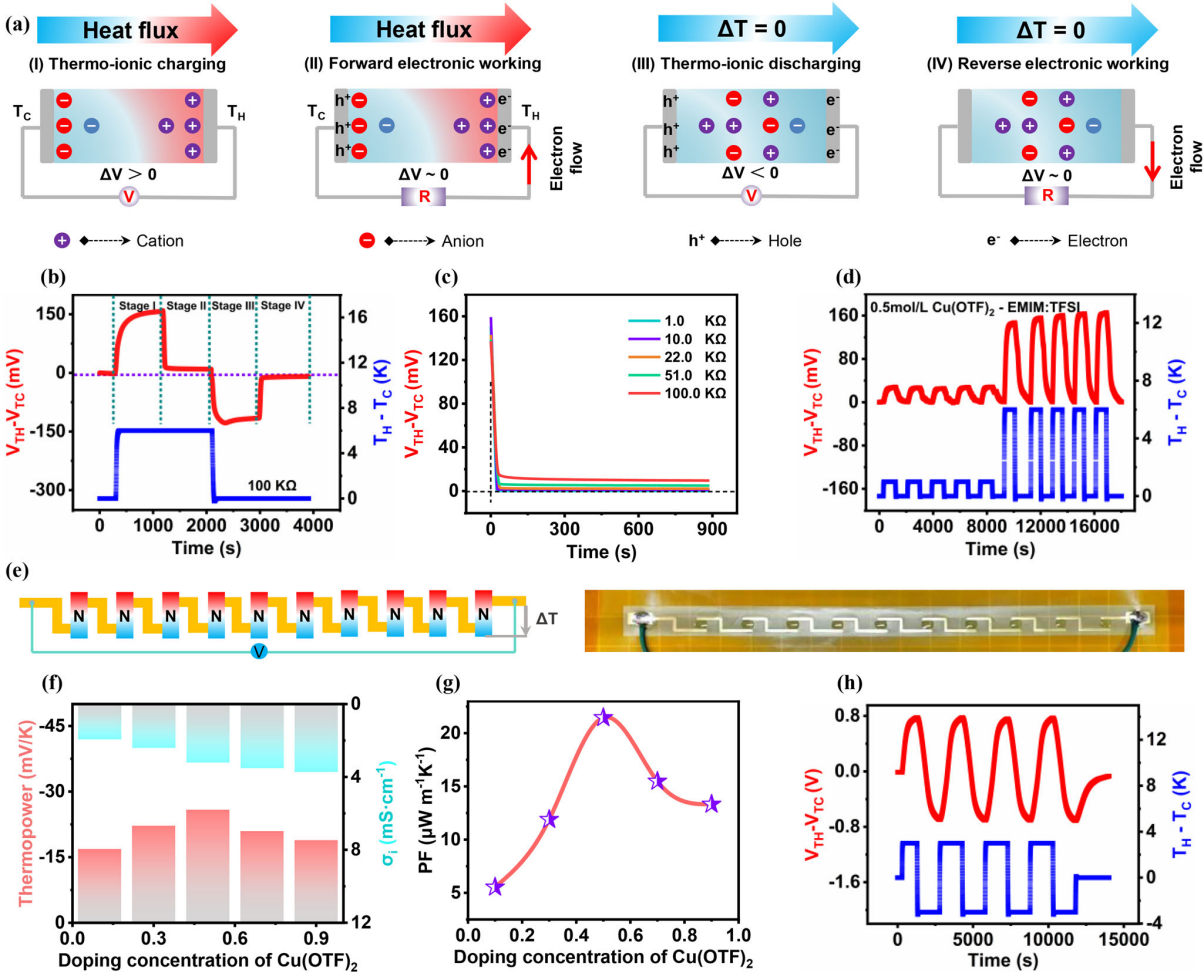
Thermoelectric properties and applications of i‐TEC and modular i‐TEC. a) Operating principle of i‐TEC. b) Charge–discharge cycle voltage curves on the external load with 100 KΩ connected to the i‐TEC. c) Voltage decay curves on the external load with different resistances connected to the i‐TEC during stage II. d) Thermal response voltage of PMEA/Cu(OTF)_2_‐EMIM:TFSI ionogel during repeated heating. e) Modular fabrication of i‐TEC with 10 n‐type thermoelectric capacitors. f) Cu(OTF)_2_ regulated thermopower of PMEA/Cu(OTF)_2_‐EMIM:TFSI ionogels. g) Power factors of PMEA/Cu(OTF)_2_‐EMIM:TFSI ionogels. h) Thermal response voltage of modular i‐TEC.

In view of the excellent ionic thermoelectric properties of the PMEA/Cu salt‐EMIM:TFSI ternary ionogel, we prepared i‐TEC devices using PMEA/Cu(OTF)_2_‐EMIM:TFSI. Two symmetric, copper gold‐plated (Au/Cu) electrodes deposited on a high‐temperature‐resistant polyimide (PI) substrate were used for the assembly of the i‐TEC device (Figure S35, Supporting Information). i‐TEC was operated in a quasi‐continuous mode, in which **Figure** **5**a denotes the operating principle of the i‐TEC and Figure 5b shows the voltage profile of the thermal response during the operation of the i‐TEC. First, the heat flow induces thermal diffusion of anions and cations through the Soret effect (Stage I). In stage I, when the temperature difference between the two ends of the i‐TEC is 6 K, the thermal voltage reaches a stable value of ≈159.6 mV after 15 min (900 s). Subsequently, when a 100 KΩ load resistor is connected, electrons flow through the external load to compensate for the charge imbalance between the two electrodes (stage II). At this stage, the thermal voltage decreases rapidly and reaches a stable value of ≈9.5 mV after 15 min. In addition, the discharge time of this stage becomes longer as the external load resistance increases (Figure 5c; Figure S36, Supporting Information). Correspondingly, we also tested the power and energy density of the i‐TEC device under different load resistances (Figure S29, Supporting Information). The test results indicate that the output power initially decays rapidly but gradually saturates to a constant value with the addition of external resistance, forming a stable thermoelectric operating mode with sustained power output maintained for 15 min. We also calculated the energy density for a series of external resistance values. The energy density exhibits a parabolic variation with a saturation value of 71.86 J m^−2^. Stage III is the thermo‐ionic discharge stage, in which the external load is disconnected and the heat source is turned off. The anions and cations gathered on the electrodes return to their original state due to the fallback of the temperature gradient. Meanwhile, due to the disconnection of the external circuit, the electrons in stage II remain on the electrode, and thus the open‐circuit voltage becomes negative (−127.1 mV) after 15 min. Finally, when the load resistor is connected again, electrons flow through the external circuit in the opposite direction. The current flow in stage IV is in the opposite direction to that in stage II. And, we also verified the pure capacitive behavior of the i‐TEC, which exhibits a nearly ideal rectangular shape in typical cyclic voltammetry test curves and symmetric constant‐current charge–discharge curves (Figure S30, Supporting Information).

We also repeatedly measured the thermal response voltage of i‐TEC during five hot and cold cycles at a temperature difference of 1 and 6 K, which is important for the repetitive and stable use of i‐TEC (Figure 5d). By adjusting the Cu(OTF)_2_ concentration, we further discussed the thermopower and ionic conductivity of the PMEA/Cu(OTF)_2_‐EMIM:TFSI ternary ionogels (Figure 5f,g; Figures S31 and S32, Supporting Information). The negative ionic thermopower of the samples first increases and then decreases with the increase of Cu(OTF)_2_ concentration. This decrease may be attributed to the increase in the viscosity of the liquid electrolyte at higher concentrations of Cu(OTF)_2_. At a Cu(OTF)_2_ concentration of 0.5 mol L^−1^, the PMEA/Cu(OTF)_2_‐EMIM:TFSI obtained a thermopower of −25.85 mV K^−1^, a conductivity of 3.21 mS cm^−1^ and a maximum of 21.45 µW/(m·K) of the power factors (PF). In addition, to further increase the output voltage signal, we demonstrated 10 i‐TECs in series as a modularity (Figure 5e). The i‐TEC module achieved an open‐circuit voltage of 0.774 V during repeated hot and cold cycling at ±3 K temperature difference, showing real‐time response to temperature gradient changes and stable thermal voltage output (Figure 5h). It also shows that at the 3 K temperature gradient, the modular i‐TEC device exhibits a short‐circuit current of 62.20 µA and an instantaneous maximum power density of 12.13 µW (Figure S33, Supporting Information). The voltage‐current curve exhibits linear variation due to high ion mobility and stability during the device's power output process. Finally, we repeatedly measured the thermal response voltage changes of the modular i‐TEC device over five days under the 3 K temperature gradient (Figure S34, Supporting Information). Test results indicate that its thermal charging voltage remained at 94.2% after five days of thermal charging. This also demonstrates that the thermoelectric device is reusable rather than a single‐use energy source.

## 3 Conclusion 

In summary, we have systematically demonstrated four generic configurations for the interconversion of anion/cation cluster structures in ternary ionogel systems based on the anion‐doped Gutmann donor theory and realised the stepwise synergistic regulation of the negative thermopower. On the one hand, the higher Gutmann DN doped anion enables it to participate in the anion cluster construction and increase the anion transport heat *Q*_−_ together with the TFSI^−^ anion due to the stronger binding energy with the doped cation. On the other hand, if the DN of the doped anion continues to increase it will inevitably lead to the creation of cation clusters with the doped anion as the coordination core and weaken the anion clusters. Our work emphasises the matching strategy of doped anions and doped cations. That is, the construction of mononuclear [Cu^2+^‐high DN doped anion] anion clusters to achieve the maximization of the negative thermopower.

Further, the systematic study in this work can be divided into 3 dimensions to specify the integrated modulation of n‐type ionic thermoelectric materials. 1) Ion‐polymer interactions in polyacrylate‐based binary ionogels modulate the thermal transport of EMIM^+^ cations and TFSI^−^ anions, respectively. Polyacrylates are typical gel‐elastomer materials, which endow i‐TE devices with excellent flexibility, self‐healing and wearability. Moreover, the molecular structure of polyacrylates is rich in a large number of oxygen‐containing functional groups and carbon skeleton structures. Synergistic modulation of the interactions between EMIM^+^ cations and polar oxygen‐containing functional groups, TFSI^−^ anions and non‐polar carbon skeletons is the first step to optimize the ionic thermoelectric properties. 2) *Q*_−_ regulation dominated by doped anions in PMEA/Li salt‐ EMIM:TFSI ternary ionogels. Among them, the negative ionic thermoelectric behavior of PMEA/LiTFA‐EMIM:TFSI is sharply curtailed due to the doping of high DN anions. We clearly observed the transition process from anionic to cationic clusters. This is an important reference for refining the theory based on the enhancement of *Q*_−_ and thermopower by strong doped ion‐ion interactions in ionic liquids. 3) Generally increase in the negative thermopower by cation doping in PMEA/Cu salt‐ EMIM:TFSI ternary ionogels. The high charge and small radius of the doped cations are the most favorable guarantees to achieve anionic clusters maximally regulating the *Q*_−_ of the doped anions and TFSI^−^ anions. Representatively, PMEA/Cu(OTF)_2_‐EMIM:TFSI ternary ionogels demonstrated a huge negative thermopower of −25.85 mV K^−1^ and a high ionic conductivity of 3.21 mS cm^−1^. In conclusion, our work provides a novel insight into the theoretical basis for designing i‐TE materials. And, the importance of conformational changes and systematic optimization of ion clusters induced by strong ionic interactions is highlighted in i‐TE materials. It has a wide range of applications in large‐scale energy harvesting and sustainable energy thermal conversion. Finally, it should be noted that this study still has certain limitations. Our work mainly focuses on specific chemical systems (such as EMIM:TFSI ionic liquid and polyacrylate matrix), and the universality of the obtained ion cluster configuration transformation rules and synergistic regulation strategies in other ionic liquids or polymer matrices still needs to be more extensively verified in future work.

## 4 Experimental Section

A detailed Experimental Section can be found in Supporting Information.

## Conflict of Interest

The authors declare no conflict of interest.

## Author Contributions

B.C., M. T., and H. W. contributed equally to this work. B.C. conceived the idea and designed the research. B.C., T.W., and C.Y. directed the whole study. B.C. and M.T. performed the majority of the experiments. B.C., M.T., Y.J., and H.T. analyzed the experimental data. H.J. and W.L. performed the DFT analysis. B.C., M.T., and Y.J. drafted the manuscript, and all authors contributed to the writing of the manuscript.

## Supporting information



Supporting Information

## Data Availability

The data that support the findings of this study are available in the supplementary material of this article.
