# Supplementary material for: Gutmann Donor Theory‐Guided Design of Mononuclear Ionic Cluster for Exceptional N‐Type Thermoelectric Ionogel
Source: Adv Sci (Weinh). 2025 Nov 21;13(7):e20143. doi: 10.1002/advs.202520143 (PMC12866749; doi:10.1002/advs.202520143)
Supplement: Supplementary file 1 — Supporting Information [file ADVS-13-e20143-s001.docx]

Supporting Information

**Gutmann Donor Theory-Guided Design of Mononuclear Ionic Cluster for Exceptional N-Type Thermoelectric Ionogel**

*Bin Chen*, Mingxuan Tian, Hongji Wang, Yun Jin, Heng Tang, Xingrui Chen, Shengxue Yu, Changyuan Yan*, Wenwei Lei*, and Taihong Wang**

*B. Chen, M. Tian, H. Wang, Y. Jin, X. Chen, S. Yu, C. Yan, W. Lei*

Hebei Key Laboratory of Applied Chemistry and Hebei Key Laboratory of Heavy Metal Deep-Remediation in Water and Resource Reuse, School of Environmental and Chemical Engineering, Yanshan University, Qinhuangdao 066004, China.

E-mail: [chenbin@ysu.edu.cn](mailto:chenbin@ysu.edu.cn) (Bin Chen), [yancy@ysu.edu.cn](mailto:yancy@ysu.edu.cn) (Changyuan Yan), and [leiww@ysu.edu.cn](mailto:leiww@ysu.edu.cn) (Wenwei Lei)

*H. Tang, T. Wang**

Department of Electrical and Electronic Engineering, Southern University of Science and Technology, Shenzhen, 518055, China.

E-mail: [wangth@sustech.edu.cn](mailto:wangth@sustech.edu.cn) (Taihong Wang).

**Experimental Section**

**Materials**

Ethylene glycol methyl ether acrylate (MEA), 2-ethoxy-ethanoacrylate (EOEA) and Ethylene glycol methyl ether methacrylate (MEMA) monomers were purchased from TCI co (Shanghai). 1-ethyl-3-methylimidazoliumbis (trifluoromethylsulfonyl) imide (EMIM:TFSI), 2-hydroxy-2-methylpropiophenone (HMPP), lithium hexafluorophosphate (LiPF_6_), lithium tetrafluoroborate (LiBF_4_), lithium trifluoromethanesulfonate (LiOTF), lithium nitrate (LiNO_3_), lithium trifluoroacetate (LiTFA), copper tetrafluoroborate (Cu(BF_4_)_2_), copper perchlorate (Cu(ClO_4_)_2_), copper trifluoromethanesulfonate (Cu(TFA)_2_), copper nitrate (Cu(NO_3_)_2_), copper trifluoroacetate (Cu(TFA)_2_) and ethanol were obtained from Shanghai Macklin Biochemical Technology Co. Ltd.

**Preparation of PMEA/EMIM:TFSI binary ionogels**

Firstly, MEA monomer, EMIM:TFSI and photoinitiator HMPP were mixed. The mass ratio of MEA and EMIM:TFSI was fixed to be 1:1. HMPP was added in the amount of 0.1 mol% to the monomer. The precursor solution was vented with nitrogen (N_2_) for 15 min and poured into a PTFE mold and sealed with a glass plate. The polymerization reaction was initiated by UV light at 365 nm for 3 h. In addition, the preparation of PEOEA/EMIM:TFSI and PMEMA/EMIM:TFSI binary ionogels can be referred to this method.

**Preparation of PMEA/Li salt- EMIM:TFSI ternary ionogels**

First, the Li salt was dissolved in ethanol according to the mass ratio 1:3. Then, the ethanol solution, MEA monomer, EMIM:TFSI and photoinitiator HMPP were mixed. Wherein, if not specified the Li salt was added in an amount of 0.5 mol/L (compared to EMIM:TFSI). The mass ratio of MEA and EMIM:TFSI remained fixed at 1:1. HMPP was added in an amount of 0.1 mol% to the monomer. Next, the precursor solution was vented with N_2_ for 15 min and poured into a PTFE mold and sealed with a glass plate. The polymerization reaction was initiated by UV light at 365 nm for 3 h. Finally, the ethanol was removed by drying overnight in a fume hood to prepare PMEA/Li salt - EMIM:TFSI ternary ionogels. The preparation of PMEA/Cu salt - EMIM:TFSI ternary ionogels can be referred to this method.

**Measurement of the thermopower**

The devices for thermopower measurements were obtained by drop-casting an ionogel precursor solution onto a PI substrate with Au/Cu electrodes deposited (thickness of the Au layer was 50 nm), and light-curing and drying as described above. Measurements of the thermopower were carried out on a homemade device along the planar direction (**Figure S35**). Two Peltiers were used to modulate the temperature gradient from 1 to 6 K. Two ultrafine K-type thermocouples were placed on the Au/Cu electrodes close to the ionogel for temperature calibration to ensure the trueness and accuracy of the temperatures. Unless otherwise stated, thermopower measurements were performed at room temperature (20 ℃) and 80% relative humidity. The humidity environment was regulated using an experimental chamber (**Figure S36**). The relative humidity of the chamber was controlled by a humidifier containing glycerol and deionized water. The relative humidity was controlled by varying the volume ratio of deionized water to glycerol in a mixed solution, which was calculated as follows^1^:

$$\frac{V_{water}}{V_{glycerol}}=1.26\left[\frac{1}{3.83\left(\left(19.9-0.189RH\right)^{0.0806}-1\right)}-1\right]$$

where $V_{water}$ and $V_{glycerol}$are the volumes of deionized water and glycerol, respectively. $RH$is the target relative humidity.

**Material characterisation**

Infrared spectra were collected using an attenuated total reflectance Fourier transform infrared spectroscopy (ATR-FTIR, Nicolet iS50 spectrometer). The Raman spectroscopy measurements of the polymer gels were performed using a Horiba Scientific LabRAM HR Evolution with 633-nm laser. NMR spectra were measured with a Bruker AVII 400 NMR spectrometer at a frequency of 400 MHz using CDCl_3_ as the solvent. Stress-strain curves were performed on a Shimadzu AGS-X tester at 10 mm/min at 25 ℃. Optical microscopy images of the self-healing process were acquired using an in-situ optical microscope (YM450TR, YUESCOPE). The resistance of the ionogel was characterized by electrochemical impedance spectroscopy (voltage amplitude of 5 mV) in the frequency range of 0.1-100 KHz using a CHI660E electrochemical workstation. The ionic conductivity was obtained by linear extrapolation. Ionic conductivity $\sigma_{\text{i}}=\frac{d}{R\times S}$, where $\sigma_{\text{i}}$ is the ionic conductivity, $d$ is the sample thickness, $R$ is the volume resistivity, and $S$ is the contact area between the sample and the electrode. All of the DFT calculations were using the Orca program package and the Electrostatic potential map (ESP) was analyzed by Multiwfn program version 3.8-dev^2, 3^.

**Figure S1**. Anion-induced conversion of anion/cation cluster structures in PMEA/Li salt - EMIM:TFSI and PMEA/Cu salt - EMIM:TFSI ternary ionogels.

**Figure S2.** Thermal response voltage and thermopower fitting curve for pure ionic liquid of EMIM:TFSI.

**Figure S3**. Fitted curves of thermal response voltage and thermopower of polyacrylate binary ionogels (PEOEA/EMIM:TFSI, PMEA/EMIM:TFSI, PMEMA/EMIM:TFSI).

**Figure S4**. DFT calculations of the binding energies for acrylate monomers with EMIM^+^ cation and TFSI^-^ anion, respectively.

**Figure S5**. Electrostatic potential diagram.

**Figure S6**. ATR-FTIR spectra of -OCH_2_CH_3_ stretching vibrational band, -HC=CH- stretching vibrational band, -S-N-S- antisymmetric bending band and -O=S=O- antisymmetric bending band in PEOEA/EMIM:TFSI binary ionogel.

**Figure S7**. ATR-FTIR spectra of CH_3_···O-CH_3_ stretching vibrational band, -HC=CH- stretching vibrational band, -S-N-S- antisymmetric bending band and -O=S=O- antisymmetric bending band in PMEMA/EMIM:TFSI binary ionogel.

**Figure S8**. H-NMR spectrum of the MEA/EMIM:TFSI mixture.

**Figure S9**. H-NMR spectrum of the EOEA/EMIM:TFSI mixture.

**Figure S10**. H-NMR spectrum of the MEMA/EMIM:TFSI mixture.

**Figure S11**. Thermal response voltage and thermopower fitting curves for PMEA/Li salt (LiPF_6_, LiBF_4_ and LiOTF) - EMIM:TFSI ternary ionogels.

**Figure S12**. Thermal response voltage and thermopower fitting curves for PMEA/Li salt (LiNO_3_ and LiTFA) - EMIM:TFSI ternary ionogels.

**Figure S13**. Raman spectra of PMEA/Li salt - EMIM:TFSI ternary ionogels.

**Figure S14**. ATR-FTIR spectra of PMEA/Li salt - EMIM:TFSI ternary ionogels.

**Figure S15**. ATR-FTIR spectra of PMEA/LiPF_6_ - EMIM:TFSI, PMEA/LiBF_4_ - EMIM:TFSI, PMEA/LiOTF - EMIM:TFSI and PMEA/LiNO_3_ - EMIM:TFSI ternary ionogels.

**Figure S16**. H-NMR spectrum in MEA monomer/Li salt - EMIM:TFSI mixtures.

**Figure S17**. Thermal response voltage and thermopower fitting curves for PMEA/Cu salt [Cu(BF4)_2_, Cu(ClO_4_)_2_ and Cu(OTF)_2_] - EMIM:TFSI ternary ionogels.

**Figure S18**. Thermal response voltage and thermopower fitting curves for PMEA/Cu salt [Cu(NO_3_)_2_ and Cu(TFA)_2_] - EMIM:TFSI ternary ionogels.

**Figure S19**. Raman spectra of PMEA/Cu salt - EMIM:TFSI ternary ionogels.

**Figure S20**. Quantitative analysis of anion cluster content in PMEA/Cu salt - EMIM:TFSI ternary thermoelectric ionogel.

**Figure S21**. ATR-FTIR spectra of PMEA/Cu salt - EMIM:TFSI ternary ionogels.

**Figure S22**. ATR-FTIR spectra of MEA/Cu(BF_4_)_2_ - EMIM:TFSI, PMEA/Cu(ClO_4_)_2_ - EMIM:TFSI、PMEA/Cu(OTF)_2_ - EMIM:TFSI and PMEA/Cu(NO_3_)_2_ - EMIM:TFSI ternary ionogels.

**Figure S23**. In-situ Raman measurement of PMEA/Cu(OTF)_2_ - EMIM:TFSI ternary thermoelectric ionogel.

**Figure S24**. The short circuit current - open circuit voltage and corresponding output power of the i-TEC constructed from the PMEA/Cu(OTF)_2_ - EMIM:TFSI ternary thermoelectric ionogel.

**Figure S25.** Charge distribution diagram of MEA and thermoelectric performance recovery rate of PMEA/Cu(OTF)_2_ - EMIM:TFSI ternary thermoelectric ionogel under 0-500% tensile strain and after 10 self-healing cycles.

**Figure S26.** Thermoelectric properties of PMEA/Cu(OTF)_2_ - BMIM:TFSI and PMEA/Cu(OTF)_2_ - EMIM:BF_4_ ternary thermoelectric ionogels.

**Figure S27**. The assembly of i-TEC.

**Figure S28**. Charge/discharge cycle voltage variation curves of i-TEC under different external loads (1 KΩ, 10 KΩ, 22 KΩ and 51 KΩ).

**Figure S29**. Power and energy density of i-TEC device under different load resistances.

**Figure S30**. Cyclic voltammetry test curves and constant current charge/discharge curves for i-TEC.

**Figure S31**. Fitted curves of the thermopower for PMEA/Cu(OTF)_2_ - EMIM:TFSI ternary ionogels by Cu(OTF)_2_ concentration.

**Figure S32**. Nyquist plots of the PMEA/Cu(OTF)_2_ - EMIM:TFSI ternary ionogels with different Cu(OTF)_2_ loadings.

**Figure S33**. The short circuit current - open circuit voltage and corresponding output power of the modular i-TEC device.

**Figure S34**. Thermal response voltage variation and performance degradation analysis of the modular i-TEC device over a 5-day period.

**Figure S35**. The illustration of the measurement setup of ionic thermopower.

**Figure S36**. Schematic diagram of the relative humidity controlled-environment chamber.

| Materials | HC=CH | NC(H)NCH | C=O | SO_2_ | CF_3_ | SNS |
| --- | --- | --- | --- | --- | --- | --- |
| IL(EMIM:TFSI) | 3159 | 3123 | - | 1348 1329 | 1226  1167 | 1050 |
| MEA | - | - | 1727 | - | - | - |
| MEA-IL | 3157 | 3118 | 1728 | 1351 1335 | 1227  1168 | 1056 |
| EOEA | - | - | 1729 | - | - | - |
| EOEA-IL | 3156 | 3116 | 1729 | 1351 1335 | 1227  1169 | 1055 |
| MEMA | - | - | 1721 | - | - | - |
| MEMA-IL | 3157 | 3117 | 1723 | 1350  1335 | 1227  1180 | 1057 |

**Table S1.** The IR band migrations of polyacrylate ionogel.

**Table S2.** Thermoelectric properties of the PMEA/Cu(OTF)_2_ - EMIM:TFSI ionogel in this study were compared with those of previously reported n-type ionogels.

| Materials | Thermopower | Relative humidity | Conductivity | Ref |
| --- | --- | --- | --- | --- |
| PVDF-HFP/NaTFSI - PC | -6 mV/K | 68% RH | 0.933 mS/cm | 4 |
| PVDF-HFP/EMIM:TFSI | -4 mV/K | 60% RH | 6.0 mS/cm | 5 |
| PVDF-HFP/LiBF_4_ - EMIM:TFSI | -15 mV/K | 60% RH | 4.5 mS/cm | 6 |
| PVDF-HFP/LiBF_4_ - EMIM:TFSI | -21 mV/K | 90% RH | 3.06 mS/cm | 7 |
| P(MMA-co-MA)/EMIM:TFSI | -8.8 mV/K | 100% RH | 3.0 mS/cm | 8 |
| PVDF-HFP/AgOTF - BMIM:BF_4_ | -26.4 mV/K | 75% RH | 13 mS/cm | 9 |
| PEO/EmimBF_4_ - LiTFSI | -20.6 mV/K | 90% RH | 1.9 mS/cm | 10 |
| PMEA/Cu(OTF)_2_ - EMIM:TFSI | -17.63 mV/K | 50% RH | 2.97 mS/cm | This work |
| PMEA/Cu(OTF)_2_ - EMIM:TFSI | -25.85 mV/K | 80% RH | 3.26 mS/cm | This work |
| PMEA/Cu(OTF)_2_ - EMIM:TFSI | -27.91 mV/K | 90% RH | 3.85 mS/cm | This work |

**References**

1. S.L. Kim, H.T. Lin, and C. Yu. Thermally Chargeable Solid‐State Supercapacitor. *Advanced Energy Materials* 2016, ***6***, 1600546. https://doi.org/10.1002/aenm.201600546.

2. F. Neese. The Orca Program System. *Wiley Interdisciplinary Reviews: Computational Molecular Science* 2012, ***2***, 73-78. https://doi.org/10.1002/wcms.81.

3. T. Lu, and F. Chen. Multiwfn: A Multifunctional Wavefunction Analyzer. *Journal of Computational Chemistry* 2012, ***33***, 580-592. https://doi.org/10.1002/jcc.22885.

4. C. Chi, M. An, X. Qi, Y. Li, R. Zhang, G. Liu, C. Lin, H. Huang, H. Dang, and B. Demir. Selectively Tuning Ionic Thermopower in All-Solid-State Flexible Polymer Composites for Thermal Sensing. *Nature Communications* 2022, ***13***, 221. https://doi.org/10.1038/s41467-021-27885-2.

5. D. Zhao, A. Martinelli, A. Willfahrt, T. Fischer, D. Bernin, Z.U. Khan, M. Shahi, J. Brill, M.P. Jonsson, and S. Fabiano. Polymer Gels with Tunable Ionic Seebeck Coefficient for Ultra-Sensitive Printed Thermopiles. *Nature Communications* 2019, ***10***, 1093. https://doi.org/10.1038/s41467-019-08930-7.

6. S. Liu, Y. Yang, H. Huang, J. Zheng, G. Liu, T.H. To, and B. Huang. Giant and Bidirectionally Tunable Thermopower in Nonaqueous Ionogels Enabled by Selective Ion Doping. *Science Advances* 2022, ***8***, eabj3019. https://doi.org/10.1126/sciadv.abj3019.

7. S. Liu, Y. Yang, S. Chen, J. Zheng, D.G. Lee, D. Li, J. Yang, and B. Huang. High P-and N-Type Thermopowers in Stretchable Self-Healing Ionogels. *Nano Energy* 2022, ***100***, 107542. https://doi.org/10.1016/j.nanoen.2022.107542.

8. W. Zhao, Z. Lei, and P. Wu. Mechanically Adaptative and Environmentally Stable Ionogels for Energy Harvest. *Advanced Science* 2023, ***10***, 2300253. https://doi.org/10.1002/advs.202300253.

9. Q. Le, Z. Chen, H. Cheng, and J. Ouyang. Giant Thermoelectric Performance of N‐Type Ionogels by Synergistic Dopings of Cations and Anions. *Advanced Energy Materials* 2023, ***13***, 2302472. https://doi.org/10.1002/aenm.202302472.

10. W. Zhao, Y. Zheng, M. Jiang, T. Sun, A. Huang, L. Wang, W. Jiang, and Q. Zhang. Exceptional N-Type Thermoelectric Ionogels Enabled by Metal Coordination and Ion-Selective Association. *Science Advances* 2023, ***9***, eadk2098. https://doi.org/10.1126/sciadv.adk2098.
